# An Anomalous Tumor

**Published:** 1885-01

**Authors:** J. McF. Gaston

**Affiliations:** Atlanta, Ga.


### THE ATLANTA MEDICAL AND SURGICAL JOURNAL.

Vol. I. JANUARY, 1885. No. 11.

### Original Communications.

### AN ANOMALOUS TUMOR.

By J. McF. GASTON, M. D., Atlanta, Ga.

The following case, embodying points of peculiar interest to the pathologist, presented no striking preliminary characteristics, nor was it accompanied by any indications of acute inflammation, and was developed in the course of three months.

At the request of Dr. J. A. Gray an examination was made on the 18th of last June of a large protuberance upon the left pectoral region in the person of Kit McIntosh, a colored man of thirty years of age, with good physique, who was employed as a laborer at one of our railroad stations, and had continued his service up to this time.

Not being able to detect any fluctuation upon a careful palpation, nor having any indication of pulsation on manipulation and auscultation, and finding a circumscribed induration which was some six inches in diameter at its base, we both inclined to the diagnosis of an adipose tumor beneath the fascia of the pectoral muscle. It was accordingly agreed to operate on the case, and I proceeded upon this view of the case on June the 20th by an incision of eight inches over the middle of the pectoralis major, in the direction of its fibres, at the line of union between the sternal attachments. This being carried through the subcutaneous cellular tissue and the muscular fascia, I encountered the fibres of the muscle, and inferred that the mass, which was still felt to be firm and resisting to the touch, might be an adenoid tumor beneath this muscle, so that I proceeded at once to make the requisite dissection to reach the lower border, which was pressing upon and bound to the serratus magnus by rigid adhesions. After separating these attachments a soft fluctuating mass was discovered beneath the pectoral muscle, which was found without pulsation, and hence free from the suspicion of having any connection with the axillary artery, so that a free incision was made into it, with the evacuation of a large quantity of sero-purulent fluid intermixed with flocculi and cheesy matter. Upon thrusting the finger into the cavity it was ascertained to have burrowed between the divisions of the pectoralis minor, and that the third and fourth ribs were entirely denuded for at least two inches of their outer surface, while the external intercostal muscles had been absorbed or disintegrated by the suppuration, and the internal intercostals gave the impression to the touch of being much attenuated, so that the wall of division of the abscess from the thoracic cavity was very thin at these points and must have soon broken down. With a view to effectual drainage another cut was made, at right angles to the previous incision, in the lower cutaneous flap, which had been dissected from the subjacent tissues, reaching down beneath the lower margin of the pectoral muscle; after which the edges of the cutaneous incisions were closed with Snowden's iron dyed silk suture, only leaving an opening at the most dependent part, from which the end of a long strip of lint left in the cavity protruded. A fold of absorbent cotton and a compress over the surface completed the dressing.

On the following day, the cavity was thoroughly washed out with a carbolized solution of iodine by a piece of tubing attached to a syringe, and a large sized drainage tube was left in the lower outlet, while india rubber adhesive plaster was secured over the entire outer surface so as to approximate the walls of the abscess and thus favor the obliteration of the cavity.

On the 22d there were less flocculi in the discharge, yet it continued of a sero-purulent nature, and though no carious bone was detected in the surfaces of the ribs that were exposed. apprehension was entertained lest there might be necrosis in connection with the disintegration and breaking down of the cell structures of the tissues. The antiseptic injection of the cavity was used as on the previous day, and the drainage tube was continued from the cavity to the opening below.

With little variation of the applications and appliances for a week, it was observed at the expiration of this time that the discharge was assuming more of the characteristics of well formed pus; and the carbolic element was dispensed with in the wash, using only an injection of the solution of iodine with iodide of potash, setting at rest all concern as to any further extension of the disintegrating process in the tissues.

The suture was now removed, indicating but little signs of suppuration in the line of the stitches, and thus confirming the claim of its manufacturers in favor of this article as a non-irritant.

After renewing the stiips over the region of the abscess and substituting the large drainage tube for a smaller one, the patient was only visited occasionally during the subsequent weeks for the purpose of injecting the antiseptic solution and cleansing the tube, and at the close of the month of July the tract of suppuration was reduced to the immediate proximity of the drainage tube, as verified by exploration; but the nature of the discharge still was not entirely satisfactory.

With a repetition of the injections twice a week during the month of August, the fistulous canal was reduced so that a shorter tube was requisite to drain it, and the external outlet had become so contracted as to render its introduction difficult, yet the discharge was of a glairy semi-opaque fluid without the proper characteristics of pus, and doubts were entertained as to the existence of necrosed bones, though no denuded surface could be detected with the probe. Dilatation was followed by lymphatitis extending to the axillary glands.

Early in September I resorted to an injection of a 10 grains to the ounce solution of nitrate of silver, and after the daily repetition without effect, the strength was doubled with very notable irritation of the surrounding textures, so that inflammation was set up in the axillary gland, which eventually terminated in suppuration and was discharged by the lancet. Poultices with mercurial and belladonna ointment, had been employed in the meantime over the entire left pectoral region, and were kept up on the axillary tumor until signs of salivation induced me to discontinue the mercurial element. As the discharge continued in the arm pit under the use of basilicon ointment, I dispensed with the tenting of the original orifice at the lower limit of the pectoral muscle during the month of October; and all the inflammatory features had so far disappeared, during the month of November, that the patient resumed his duties as a laborer at the railroad depot. A slight watery fluid still escaped from the fistulous opening and there was some purulent discharge from the orifice in the axillary region, but entirely without pain or other inflammatory indication, so that he was dismissed from further treatment or observation.

Now on December 8th, 1884, the patient presents himself without discharge from either orifice, and there is scarcely any remnant of the induration of the tissues in that region. This circumstantial detail of facts connected with the case leaves the reader to form his own judgment of its nature; but it is requisite to state that there is no history of local injury, either by contusion or from muscular lesion by over exertion, nor is there any trace of a general cachexy which may give a clue to its origin. The appearance of the discharge corresponded in some respects to that which is observed in cold abscess of a scrofulous character, or to that of the so-called tuberculous abscess, yet the absence of concomitant symptoms indicating any constitutional taint militates against this solution. Necrosis of bone is set aside by its termination without exfoliation. The extension of inflammation to the axillary glands is accounted for by the local irritation of the mechanical dilatation of the outlet of the abscess and the chemical action of the injection in the fistulous tract, being extended through the lymphatics. The tumor did not involve the rudimentary glandular structure of the mammary region, being entirely above the nipple of the breast, and hence not to be confounded with tubercle of the male breast, described by Dr. Poirier as occurring in Lariboisiere Hospital in 1881, and by Dr. Horteloup in a case of earlier date at Paris.

The induration and thickening of the tissues over and around the abscess disguised its character so completely that the examination by an exploring needle was not resorted to, and had it been introduced at the most salient point of the protuberance would not have been carried to a sufficient depth to reach the fluid beneath the gross fibres of the muscle; but if the true character of the case had been comprehended, a trocar might have readily been passed beneath its lower border into the cavity of the abscess.

I may state as a sequel to this rare case, that upon the occasion of making my examination in this case to-day, the patient exhibited his little son of three years of age with an induration and thickening of the structure on the right side of the breast corresponding almost to the protuberance on the left side presented originally by the father. With the concurrence of Dr. K. C. Divine, who saw the case with me, I passed an exploring needle to the depth of an inch and a half under the lower and outer part of the pectoralis major, but without finding any cavity or discharging any fluid. This has developed within a few weeks, and without any history of local injury; so that it may throw some light in its further progress upon the nature of the father's case connected with a strumous diathesis. As a resolvent application I prescribed as follows:

| | |
|---|---|
| ℞—Mercurial ointment, . . . . . | *aa.* |
| Belladonna ointment, . . . . . | ℥ss. |
| Iodine, . . . . . . . . . . | 10 grs. |
| Iodide of potash, . . . . . . . | ʒss. |

Mix and rub over the swelling, night and morning. I will watch this case and report the result at a future day.

In attempting any elucidation of the modifications observed on the part of of the tissues involved by this disintegrating process as a result of inflammatory action, I may be allowed to present some general considerations touching the fundamental principles of pathology, for which I ask the indulgent consideration of those who may be disposed to study this matter.

The abnormal changes in the superficial and deep-seated parts with their corresponding developments, call for our attention. Preliminary to general structural diseases and underlying the local disorders of vital structures there is a derangement of tissue involving the vascular network which enters into the composition of the various organs. This consists in a disturbance of the ordinary relations of a part to the whole organization, dependent upon a want of equilibrium in the circulation of the blood, and is known by the term inflammation. The harmonious co-operation of the central apparatus and the outer conduits concerned in the circulation of the blood is requisite for health, and anything which tends to interrupt this correspondence in the operations of the inner and remote parts of the machinery constitutes disease.

This disturbance may result from a local injury, impairing the proper performance of the office pertaining to the elements which make up the tissues of any part of the body, or it may ensue from a perturbation of a widespread order being concentrated upon a special region and inducing irregularity in a circumscribed area. While structural changes supervene in either case, the origin of the inflammation may be attributed to the impression received by the minute nervous distribution to the tissues involved, and whether the lesion be from a blow, an incision, or a cautery, the influence must be first transmitted to the central portion of the nervous system and returned by a line of communication to the part, for the effect to be produced in such locality. The same kind of transmission is requisite for the local manifestation of general causes which operate from without or from within the body. Any development of the inflammatory process depends upon the vital properties of the tissues, and is usually an effort of the organism to overcome an injurious impression on the constituents of the tissues which enter into the composition of the organ or part involved. The final cause of inflammation is clearly recuperation of the lost vigor in the capillaries, and though resolution or reconstruction may not be effected, the end and aim of the various steps observed in this reactionary movement must be considered as conservative. When the result is destructive, inflammation has failed in the attainment of its legitimate object, and hence Agnew classes it under the heads of healthy and unhealthy, considering inflammation as a form of hypernutrition.

As the general system, when subjected to the influence of depressing agencies, manifests an increased activity for the restoration of its normal state, and thus goes beyond the ordinary standard of excitement, so in the impairment of the vigor in certain parts there is an augmentation of the powers in the effort to regain the natural state, and we find that combination of redness and swelling with heat and pain, which characterize the inflammatory process. This new form of life comes from determination, congestion, and stagnation of the increased supply of the blood to the part. The time-honored principle of where there is irritation, to this point a flow occurs, expressed in the laconic Latin phrase "*ubi irritatio, ibi fluxus,*" gives a key to the train of sequences from any injurious impression upon the tissues, and whatever may be the explanation of the transformation which is set up in the integral elements, there is no question in regard to the determination or increased supply of blood to the part in the incipient stage of inflammation. The vascular turgescence is accompanied by that condition known as congestion, which is simply a damming up partially of the outlet of the blood, while stagnation ensues from the occlusion of the capillary vessels that transfer the blood from the arteries to the veins. From this accumulation of blood in the part, a deposition takes place in a way that the interstices of the minute interlacing of the terminal branches of the arteries and veins are filled up, so as to increase the bulk and consistence of the affected area in that characteristic form styled swelling. Up to this point in the progress of inflammation, there is accretion of substance, and it would seem that there was an effort of nature to remedy the local injury, so that we may properly designate this as a conservative process which may result in the restoration of the normal state of the tissues by what is styled a resolution, or may lead to suppuration in which a drain from the superabundant material reduces the mass to a natural consistence and a proper performance of its functions in co-operation with the adjacent structure, thus correcting the hyper-nutrition.

The most comprehensive division of inflammation is into the acute and chronic forms, or, in other words, the fast and slow processes, as this is really the only groundwork for such a line of separation. The rapid development and decline of the acute form of inflammation may depend upon the activity of the vital functions, or a sthenic condition of the system, without any characteristic difference in the commencement or progress of the perverted vascular action. It is a distinction in degree rather than in kind, and calls for measures of treatment corresponding to this phase of activity or energy in the tissues involved.

That inflammation undergoes certain modifications, according to the nature of the structure that is involved, does not materially change the true elementary principles involved in the process, and though all will understand that acute diseases, such as phlebitis, lymphatitis, peritonitis, meningitis, parotitis, orchitis, etc., with chronic affections, such as thisis pulmonalis, scrofula, secondary and tertiary syphilis, call for different treatment, yet the primordial element of perverted vascular action in the initiatory derangement presents very similar conditions in the various tissues. The commencement of such disturbance has been a subject of the closest investigation, and while certain definite changes in the arrangement of the blood corpuscles, from their normal relations to the minute vessels, have been verified, there is still a difference in the views of scientific experimenters as to the exact mode by which the result is accomplished in the exudation of a portion of the constituents of the blood.

The immediate and primary development of the first process of inflammation is accompanied by very minute leucocytes, which are held by some to be from germination in the tissues, and by others owing to migration from the blood. In the arrest of the circulation which occurs in an inflamed part, it appears that the colored corpuscles become crowded together in the capillaries, while the liquor sanguinis escapes into the interstitial reticular space. Some believe that the accumulation of the red corpuscles is due to a property they have within themselves of cohering together, owing, however, to the condition of the vessels. This is confirmed by the experiments of substituting milk or the defibrinated blood of animals for the circulating fluid, and by introducing substances which impaired the vitality of the coats, when no stasis ensued from irritation in repeating the experiments.

As to the further progress of inflammation in which a purulent degeneration exists, there have been like differences in the results of the investigations. While exudation of serum may occur through infinitely small apertures in the vascular walls, there is not such a metamorphosis of tissue as takes place in the formation of pus. The microscope has not been able to make out a distinction between the pus-corpuscle, the mucous corpuscle, and the white blood corpuscle; and there is some ground to believe that the pus cells correspond to the white corpuscles of the blood. There is a marked difference in the purulent proclivity on the part of various tissues, and under varying circumstances, that surround the case. While suppuration sets up readily in the inflammation of mucous membranes and in cellular tissues, it is slow to be developed in serous membranes and in the fibrous tissues, or in cartilage.

It might be readily comprehended that the subjacent cellular tissue would undergo such degeneration as to lead into the kind of suppuration observed in this case, if there appeared any sufficient cause for the initiatory steps of inflammation. And proceeding upon the principle of exclusion, I cannot discover in any of the ordinary excitants or disturbing elements a proper origin of the trouble, so that my mind has turned to a source which at least is not more improbable than the supposition that micrococci had penetrated to this hermetically-sealed cavity with their wonder-working transformations of tissue.

After conferring with my colleague, Dr. Gray, who has co-operated with me, not only in the original treatment, but in the subsequent management of this protracted disorder, as to the class of diseases to which our case should be assigned, without reaching any satisfactory solution, I have, upon my individual responsibility, fallen back into the obscure resources of pathology for explaining the hidden cause of disease by the recognition of embolism. I confess that this view is adopted somewhat on the principle that the backwoodsman recommended his "'possum dog," having tried him for everything else without discovering any hunting qualities in him; yet the more my thoughts are directed to the underlying principles which should guide us in diagnosis, and the more I have investigated authorities, I become deeper impressed with the conviction that some such transformation as that which results from thrombus may have been the initiatory stage of trouble in this very peculiar abscess.

In the recent work of Prof. Ziegler,on pathological anatomy, it is stated that "we may have l calized death or necrosis in a solid tissue and we may likewise have a localized death of the blood. The retrogressive and formative changes in cells and inter-cellular substances which we have recognized as the manifestation of diseased functions in the solid tissues, have their analogies in the elements of the blood." "Thrombosis may occur in any part of the vascular system." "The first result of the lodgment in the vessel or *embolism* is, that fresh deposits of fibrin take place on the plug, so that it soon occludes the vessel completely, even if too small to do so originally." "After some weeks or months the site of the embolus is often marked by nothing more than a fibrous band, or a nodular protuberance on the inner coat of the vessel. In other instances the lumen is crossed by numerous threads running singly or connected into a loose network. But the process is necessarily very different when the embolus sets up destructive inflammation around it. The inflammation then takes the suppurative form, the vessel wall, the sheath and the surrounding tissues are successively attacked, and an embolic abscess is formed." "The most unfavorable issue of all is the puriform or yellow softening of the thrombus. In this case the thrombus is transformed into a dirty or reddish yellow fetid pus-like cream or pulp."

If the absence of any predisposition to disease on the part of our patient is considered in connection with the want of data as to any exciting cause, my colleagues may not look upon this interpretation as far-fetched or unreasonable. But to those who do, I appeal for a more plausible explanation.

